# Quality of work life and professional ethics among occupational health professionals: a social exchange theory perspective

**DOI:** 10.1186/s40359-026-05226-6

**Published:** 2026-07-22

**Authors:** Omid Akbarzadeh, Roghayye Rasouli, Parisa Moshashaei, Rasoul Ahmadpour, Neda Gilani, Mehran Seif Farshad, Seyyed Shamseddin Alizadeh

**Affiliations:** 1 Department of Occupational Health & Safety, Faculty of Health, Tabriz University of Medical Sciences, Golgasht St, Tabriz, Iran; 2 School of Built Environment and Design, University of Canberra, Canberra, Australia; 3 Department of Basic Sciences, School of Allied Medical Sciences, Medical Education Research Centre, Tabriz University of Medical Sciences, Tabriz, Iran

**Keywords:** Professional ethics, Quality of work life, Occupational health professionals, Social exchange theory, Workplace ethics

## Abstract

**Background:**

Despite the increasing complexity of ethical challenges in occupational health settings, limited attention has been paid to the ethical conduct of occupational health professionals themselves. This study examined how Quality of Work Life is associated with professional ethics among occupational health professionals, guided by Social Exchange Theory.

**Methods:**

A cross-sectional survey was conducted among 200 occupational health practitioners in East Azerbaijan. Data were analysed using SPSS and SmartPLS, involving Pearson correlation, t-tests, ANOVA, and structural equation modelling. Measurement models were validated for reliability, convergent and discriminant validity, and common method bias.

**Results:**

The findings showed a statistically significant but modest positive association between the total Quality of Work Life score and the total Professional Ethics score (*r* = 0.281, *p* < 0.01). The strongest domain-level correlation was observed between Human Capabilities Development and Professional Autonomy (*r* = 0.330, *p* < 0.01). Human Capabilities Development, Safe Work Environment, and Social Integration were positively associated with Professional Responsibility, Professional Autonomy, and Trustworthiness. However, the model explained only 21.4% of the variance in Professional Responsibility, 18.6% in Professional Autonomy, and 8.2% in Trustworthiness, indicating modest explanatory capacity and particularly limited explanatory power for Trustworthiness.

**Conclusion:**

The findings suggest that Quality of Work Life may be a relevant organizational factor associated with professional ethics in occupational health practice. The study highlights the potential importance of developmental and relational aspects of work in supporting moral responsibility, while acknowledging that causal conclusions cannot be drawn from the cross-sectional design. These findings are especially relevant for policymaking in developing countries where ethical conduct is central to workplace safety and public health credibility.

**Graphical Abstract:**

**Figure:**
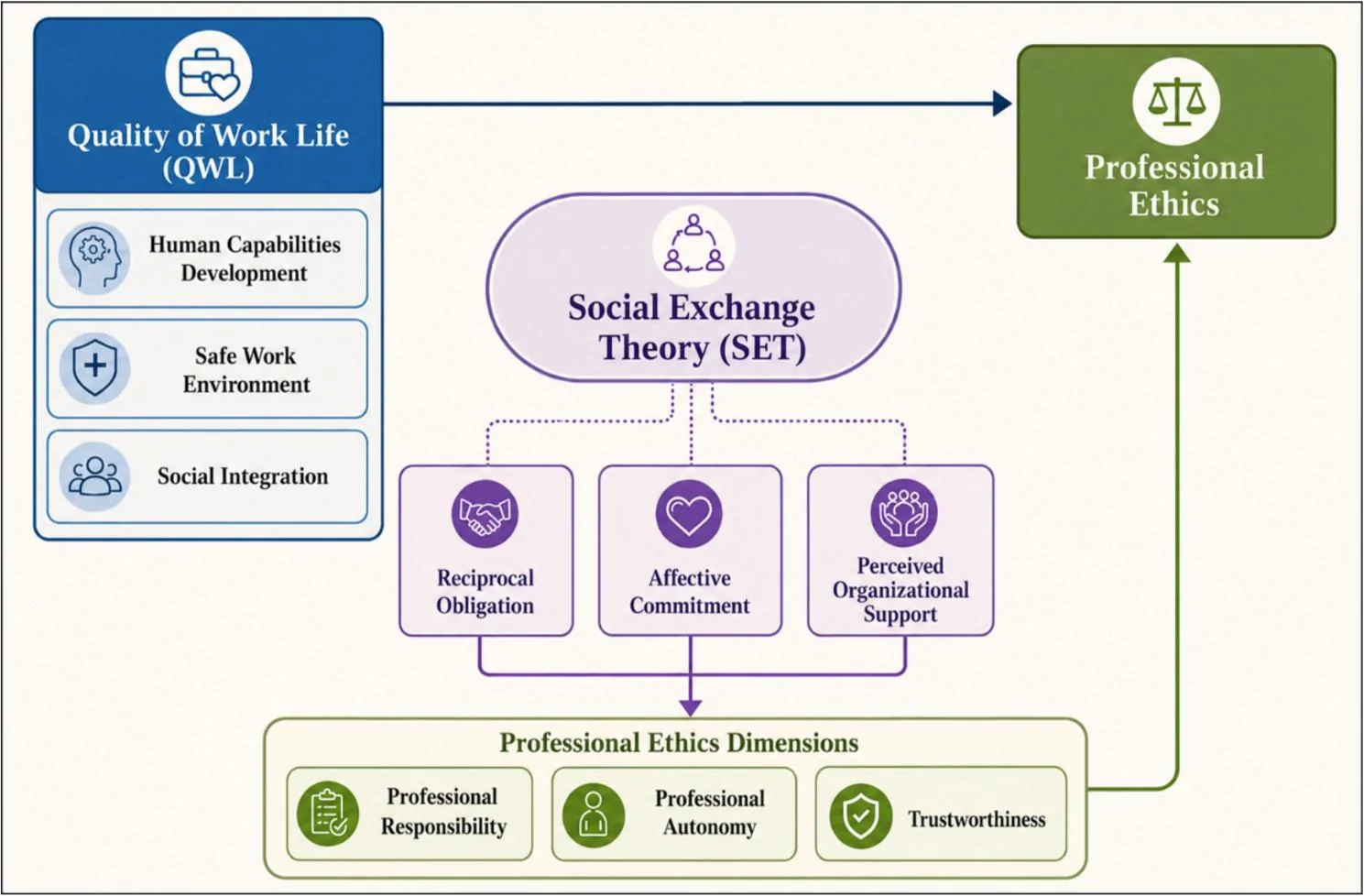

**Supplementary Information:**

The online version contains supplementary material available at https://doi.org/10.1186/s40359-026-05226-6.

## Introduction

### Background

Professional ethics has become an increasingly important concern in modern organizations, particularly in sectors where professional decisions directly affect human health, safety, and organizational legitimacy. Ethical conduct is no longer limited to individual morality or normative professional codes; rather, it is shaped by the interaction between personal values, occupational responsibilities, organizational expectations, and institutional constraints [1, 2]. This issue is especially important in workplace health and safety (WHS), where ethical failures may expose workers to preventable harm, weaken trust in safety systems, and compromise the credibility of occupational health practice [3, 4]. Occupational health professionals often operate in an “interpretative space” in which they must balance their responsibility to protect workers with organizational pressures, resource limitations, and regulatory expectations [5]. Therefore, understanding the organizational conditions that support or weaken ethical behaviour among these professionals is an important research and practical priority.

Quality of Work Life (QWL) has been widely recognized as a multidimensional organizational construct that reflects the extent to which the work environment supports employees’ physical safety, psychological wellbeing, autonomy, development, fairness, and social integration [6–8]. In occupational settings, QWL is particularly relevant because employees’ professional conduct is shaped not only by formal rules but also by the quality of the environment in which they perform their duties. Supportive working conditions, fair treatment, safe work environment, opportunities for development, and constructive interpersonal relations may be associated with employees’ motivation, psychological resilience, and sense of professional responsibility [9]. Evidence from WHS-related contexts also suggests that organizational support, interpersonal trust, and psychosocial conditions influence safety-related behaviours, including compliance with protective practices and adherence to professional standards [10].

The relationship between QWL and professional ethics is particularly important among occupational health professionals because their work involves both technical and moral responsibilities. These professionals are expected to assess risks, protect worker wellbeing, communicate hazards honestly, and support compliance with occupational health and safety regulations. However, their ethical behaviour may be affected by the organizational conditions in which they work. Poor QWL, including limited development opportunities, unsafe or stressful working conditions, weak social integration, or lack of organizational support, may reduce professional motivation and weaken ethical commitment. Previous studies have shown that diminished QWL is associated with lower job satisfaction, higher burnout, and weaker professional commitment, particularly in high-pressure healthcare and industrial environments [11, 12]. These findings suggest that QWL may be meaningfully linked to ethical behaviour, but this relationship requires clearer theoretical and empirical examination among occupational health professionals.

### Literature review

Previous research on professional ethics has examined ethical behaviour from several theoretical and empirical perspectives. Some studies have focused on the role of professional codes, ethical awareness, and formal ethics training, while others have emphasized the influence of organizational climate, leadership, institutional support, and peer norms. For example, Adams et al. showed that rapid technological and organizational changes can disrupt established ethical norms and create new ethical challenges [13]. Nikolaidis et al. argued that formal ethics enculturation alone may be insufficient to ensure ethical conduct in complex and dynamic work environments [14]. Similarly, Dejean et al., in a national study of medical physicists, highlighted the importance of institutional support and professional norms in sustaining ethical standards. These studies indicate that professional ethics is not only an individual-level characteristic but is also shaped by the broader organizational and social environment [15].

In healthcare and human-service settings, professional ethics has also been linked to important workplace outcomes. Asadi et al. examined the relationship between resilience and professional ethics among nurses in southern Iran and reported a statistically significant association between the two constructs [16]. Razavi et al. found that adherence to ethical principles among Iranian dentists was related to higher patient trust and satisfaction [17]. In addition, Asadi et al. found that resilience was associated with ethical commitment among nurses, suggesting that personal and contextual resources may influence professionals’ ability to maintain ethical conduct under occupational stress [16]. These findings collectively show that ethical behaviour is affected by both individual capacities and workplace conditions.

A growing body of organizational research has further emphasized the role of ethical leadership, workplace climate, perceived fairness, and organizational support in shaping ethical behaviour. Jha and Singh suggested that ethical behaviour may be understood as a response to perceived organizational justice and managerial sincerity, particularly when employees believe that their contributions and wellbeing are valued [18]. Mehra and Narwal found that employees’ ethical behaviour was shaped by ethical leadership and workplace climate [19]. Sha et al., drawing on Social Exchange Theory and affective-cognitive perspectives, showed that supportive and morally consistent organizational systems were associated with lower levels of unethical workplace deviance [20]. Similarly, studies demonstrated that ethical climate and leadership conditions influence employee behaviour through mechanisms of justice and confidentiality, trust, and perceived integrity. These studies support the view that ethical behaviour is co-produced through individual, interpersonal, and organizational mechanisms [21, 22].

Quality of Work Life provides a useful framework for examining these organizational and psychosocial conditions. QWL reflects the degree to which the workplace provides employees with safe conditions, opportunities for growth, social support, justice and confidentiality, professional autonomy, and meaningful participation [23]. Prior research has shown that QWL is associated with job satisfaction, organizational commitment, burnout, wellbeing, and work-related performance [7, 11]. From an ethical perspective, QWL may also influence whether professionals feel supported, respected, and morally obligated to act in accordance with professional standards. When employees experience fair treatment, safe conditions, opportunities for development, and positive social relationships, they may be more likely to reciprocate through responsible, autonomous, and trustworthy professional behaviour.

Social Exchange Theory (SET) provides a coherent theoretical foundation for explaining this relationship. SET proposes that workplace relationships are shaped by reciprocal exchanges between employees and organizations. When employees perceive that the organization provides support, fairness, respect, and resources, they are more likely to respond with positive attitudes and behaviours [24]. In this framework, QWL can be understood as an organizational resource offered to employees, while ethical behaviour can be interpreted as one possible form of reciprocal professional response. Dimensions such as human capabilities development, safe working conditions, and social integration may signal that the organization values employees and supports their professional role. In return, employees may show stronger professional responsibility, greater Professional Autonomy, and higher trustworthiness. This interpretation is consistent with previous studies showing that organizational support, ethical climate, justice and confidentiality, and perceived respect are associated with ethical conduct and professional commitment [18, 19].

**Fig. 1:**
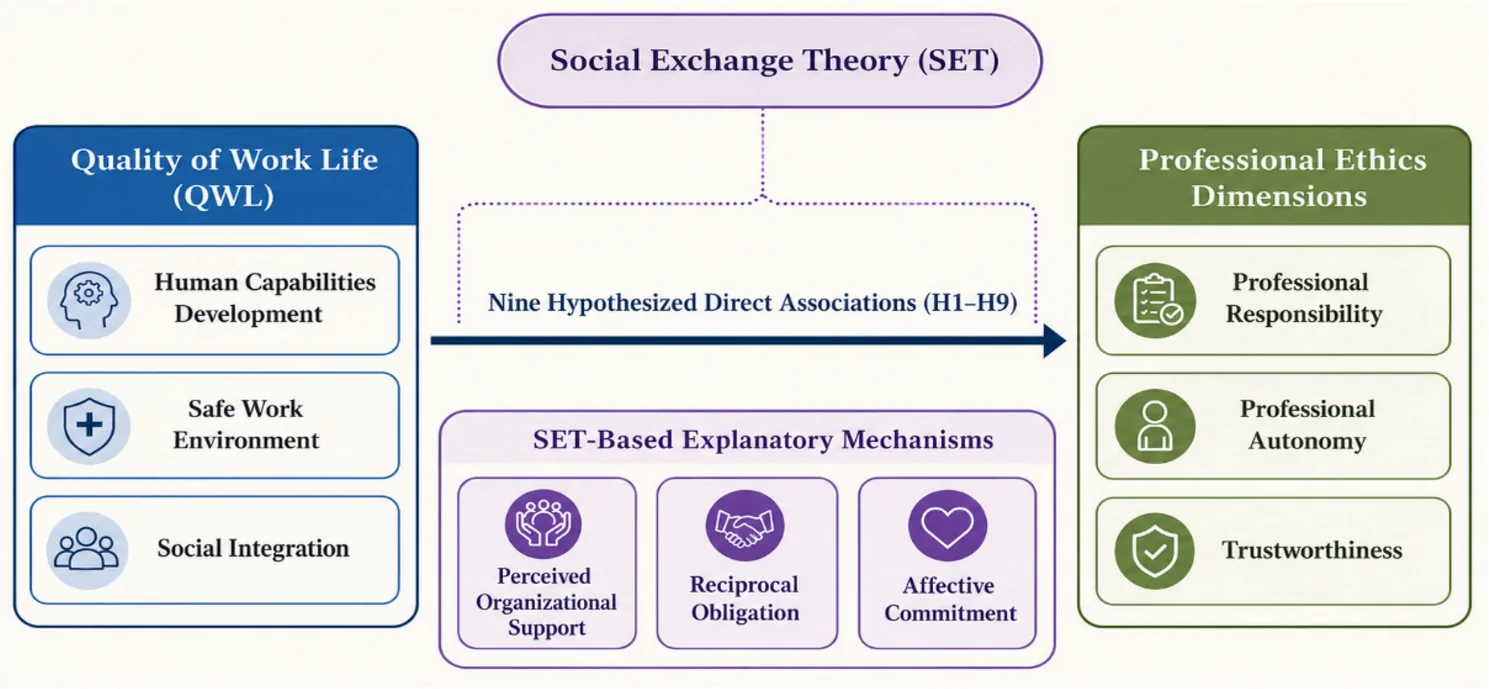
Proposed conceptual model of QWL–ethics linkage based on SET

Based on the reviewed literature and SET, the proposed conceptual framework assumes that selected QWL dimensions, including human capabilities development, safe work environment, and social integration, are positively associated with professional ethics dimensions, namely professional responsibility, professional autonomy, and trustworthiness (Fig. 1).

Despite these contributions, important gaps remain in the literature. First, many previous studies have examined either QWL or professional ethics separately, while fewer have examined how specific dimensions of QWL are associated with specific dimensions of professional ethics. Second, much of the existing evidence relies on descriptive statistics, simple correlations, or regression-based approaches, which may not fully capture the multidimensional relationships among latent organizational and ethical constructs. Third, occupational health professionals remain underrepresented in this line of research, despite their important role in protecting worker health, supporting safety compliance, and managing ethical tensions between workers, employers, and regulatory expectations. Therefore, further empirical work is needed to clarify how key dimensions of QWL are structurally associated with professional ethics among occupational health professionals. To address this gap, the present study uses Partial Least Squares Structural Equation Modelling (PLS-SEM) to examine the associations between selected QWL dimensions and professional ethics dimensions within a Social Exchange Theory framework.

### Research hypotheses

Grounded in Social Exchange Theory, this study assumes that favourable work conditions may be associated with stronger professional ethics because employees who perceive support, justice and confidentiality, safety, and respect in the workplace are more likely to respond with positive professional attitudes and behaviours. Accordingly, three dimensions of QWL were selected as key organizational resources that may be linked to three dimensions of professional ethics: professional responsibility, professional autonomy, and trustworthiness. Human capabilities development reflects opportunities for learning, skill use, and professional growth. Previous studies indicate that professional development, perceived organizational support, and empowerment are associated with stronger work engagement, ethical commitment, professional responsibility, and responsible professional conduct [18, 23]. These findings provide empirical support for expecting human capabilities development to be associated with professional responsibility, professional autonomy, and trustworthiness among occupational health professionals. Therefore, the following hypotheses are proposed: H1: Human Capabilities Development is positively associated with professional responsibility. H2: Human Capabilities Development is positively associated with professional autonomy. H3: Human Capabilities Development is positively associated with trustworthiness.

Safe work environment reflects physical safety, psychological support, and protection of employee wellbeing. In occupational health settings, a safe and supportive workplace may reinforce consistency between organizational values and professional ethical standards, thereby supporting responsible, autonomous, and trustworthy conduct [7, 12]. This evidence supports the expectation that safe work environments may be linked to greater professional responsibility, professional autonomy, and trustworthiness. Accordingly, the following hypotheses are proposed: H4: Safe Work Environment is positively associated with professional responsibility. H5: Safe Work Environment is positively associated with professional autonomy. H6: Safe Work Environment is positively associated with trustworthiness.

Social integration refers to respectful relationships, cooperation, inclusion, and trust within the workplace. Existing studies suggest that interpersonal trustworthiness, ethical climate, social support, peer norms, and workplace inclusion are associated with ethical behaviour, professional commitment, honest communication, and responsible conduct [15, 19]. These findings justify the expectation that social integration may be positively associated with professional responsibility, professional autonomy, and trustworthiness. Thus, the following hypotheses are proposed: H7: Social Integration is positively associated with professional responsibility. H8: Social Integration is positively associated with professional autonomy. H9: Social Integration is positively associated with trustworthiness.

## Methodology

### Research design

This study employs a quantitative, cross-sectional research design to empirically examine the structural relationship between QWL and Professional Ethics among occupational health professionals. The design was selected to capture real-time perceptions and behaviours in industrial settings where ethical performance may be linked to organizational context. A cross-sectional approach is particularly suitable for theory-driven modelling studies that focus on testing associations among latent constructs at a specific point in time, rather than inferring causality or temporal change. Moreover, this design enables efficient data collection in occupational environments where access to professionals is often limited, and longitudinal follow-up is not feasible.

The conceptual model is grounded in SET, which posits that employees’ attitudes and behaviours are shaped by the perceived reciprocity in their relationship with the organization. Within this framework, the various dimensions of QWL function as exogenous predictors, while components of professional ethics serve as endogenous outcomes. The model assumes that when employees perceive fairness, support, and opportunities for growth, they are more likely to demonstrate ethically aligned behaviours at work. To test this multidimensional theoretical structure, the study utilizes PLS-SEM. PLS-SEM was selected because it is suitable for estimating complex latent-variable models [25], with moderate sample sizes and non-normal data. Its use in the present study was intended to examine the proposed associations rather than to claim strong predictive performance [26].

### Population and sampling

The study population consisted of occupational health professionals employed in medium- and large-scale industrial enterprises in East Azerbaijan Province, Iran. A census sampling strategy was employed due to the relatively small, well-defined, and accessible population of occupational health professionals within the selected industrial settings. This approach was chosen to maximize representativeness, reduce sampling bias, and ensure sufficient statistical power for PLS-SEM analysis. Given the specialized nature of the target group and their limited population size, probability-based sampling techniques were not considered practical or methodologically advantageous in this context.

A total of 230 eligible individuals were invited to participate. After screening the returned questionnaires, 30 responses were excluded due to incompleteness or inconsistent responses. Therefore, the final analytic sample consisted of 200 valid responses, corresponding to an effective response rate of 86.9%. Inclusion criteria were: (1) formal employment in an occupational health role (2), a minimum of one year of continuous work experience in the current setting, and (3) active involvement in WHS-related tasks such as risk assessment, compliance training, or health surveillance. The unit of analysis was the individual professional, and the unit of observation was the self-reported response to standardized questionnaires on QWL and professional ethics. This approach ensured that inferences were drawn at the individual level rather than institutional aggregates.

Respondents were drawn from petrochemical, mining, manufacturing, and food processing sectors. The gender distribution was relatively balanced, with a slightly higher proportion of female participants (53.5%) than male participants (46.5%). The dominant age group was 20–30 years, representing 63.5% of the sample, followed by those aged 31–40 years (27.5%). Most participants had 1–10 years of professional experience, and 71.5% held a bachelor’s degree.

Regarding sample adequacy for PLS-SEM, the final analytic sample size of 200 met the minimum requirements based on both the “10-times rule” and statistical power criteria recommended by Hair et al. [26]. Specifically, the model includes a maximum of six paths leading to any endogenous construct; therefore, the minimum suggested sample size is 10 × 6 = 60 observations. With 200 valid cases, the dataset satisfies the minimum criteria for path coefficient estimation, model convergence, and hypothesis testing in PLS-SEM analysis.

### Instrumentation

This study employed a structured questionnaire composed of two psychometric tools: a standardized QWL instrument and a researcher-developed Professional Ethics scale. Both instruments were adapted and validated for the context of occupational health professionals and were designed for reflective measurement modelling in PLS-SEM analysis. Examples of questionnaire items and their corresponding conceptual dimensions are provided in Supplementary File 1.

#### Work life quality instrument

The QWL construct was measured using a Persian-translated version of Walton’s model [27], comprising 27 items across eight dimensions: Fair Compensation, Safe Work Environment, Growth Opportunities, Organizational Constitutionalism, Work-Life Balance, Social Relevance, Social Integration, and Human Capabilities Development. All items were rated on a five-point Likert scale (1 = Strongly Disagree to 5 = Strongly Agree), and all were positively worded. No reverse-coded items were included. Final scores were computed using arithmetic means for each dimension and the total construct.

The content validity of the QWL instrument was assessed by a 10-member expert panel evaluating item necessity, relevance, clarity, and contextual appropriateness. The dimension-level CVR and I-CVI values are presented in Table 1 and met the acceptable criteria. Minor wording modifications were made, but no items were removed.

**Table 1:** Content validity results for the quality of work life instrument

QWL dimension	Number of items	Mean CVR	Mean I-CVI
Fair Compensation	4	0.92	0.94
Safe Work Environment	4	0.88	0.89
Growth Opportunities	3	0.93	0.95
Organizational Constitutionalism	3	0.81	0.85
Social Relevance	3	0.87	0.90
Work-Life Balance	4	0.94	0.96
Social Integration	3	0.84	0.88
Human Capabilities Development	3	0.90	0.92
Total QWL instrument	27	0.89	0.91

To explore the factorial structure of the QWL instrument, EFA was conducted in SPSS using principal component extraction with Varimax rotation. Sampling adequacy was supported by a KMO value of 0.91 and a significant Bartlett’s test of sphericity (χ² = 2984.12, *p* < 0.001). Items with factor loadings ≥ 0.60 and eigenvalues > 1 were retained. The reflective measurement model was subsequently evaluated in SmartPLS 4 using standardized factor loadings, composite reliability, AVE, and HTMT. The measurement-model results are reported in "Measurement model evaluation" section.

#### Professional ethics instrument

The professional ethics questionnaire was a researcher-developed tool based on a synthesis of existing national professional ethics codes, guidelines published by Iran’s Ministry of Health [28], and previous literature [16]. It included 29 items across six conceptual domains: professional excellence (3 items), trustworthiness (5 items), Service Quality (3 items), Professional Autonomy (4 items), professional responsibility (8 items), and justice/confidentiality (6 items). All items used a five-point Likert scale (1 = Strongly Disagree to 5 = Strongly Agree), with no reverse-worded items.

The content validity of the tool was assessed by a panel of 10 experts using Lawshe’s method. The CVR values ranged between 0.88 and 0.94, and the CVI ranged between 0.92 and 0.97 (Table 2). In addition, interpretive validity was addressed by asking three domain specialists to review the items for alignment with the intended ethical constructs in clinical and occupational health settings.

**Table 2:** Content validity metrics (CVR and CVI)

Construct	Content Validity Ratio (CVR)	Content Validity Index (CVI)	Cronbach’s Alpha
Quality of Work Life (Total)	0.89	0.92	0.89
Professional Excellence	0.91	0.95	0.83
Trustworthiness	0.90	0.94	0.87
Service Quality	0.92	0.96	0.85
Professional Autonomy	0.94	0.97	0.86
Professional Responsibility	0.88	0.93	0.91
Justice & Confidentiality	0.90	0.94	0.88

The Professional Ethics instrument was evaluated using the same EFA and reflective measurement-model procedures described in "Data analysis strategy" section. The corresponding factor-analytic results are reported in "Measurement model evaluation" section.

#### Common method bias

The questionnaires were administered in Persian using a forward–backward translation procedure conducted by two bilingual experts. Cultural suitability was further assessed through a pilot test with 20 occupational health professionals, confirming item clarity, relevance, and readability. Common method bias was examined using Harman’s single-factor test and measurement-model VIF values. These procedures were treated as diagnostic checks rather than definitive evidence of the absence of common method bias.

### Data analysis strategy

Data were analysed using SPSS and SmartPLS 4 through four sequential stages. First, the dataset was screened for incomplete responses, missing values, distributional characteristics, and extreme outliers. Descriptive statistics were then calculated, followed by independent-samples t-tests, one-way ANOVA, and Pearson correlation analysis as supplementary procedures for examining group differences and bivariate associations. 

Second, the psychometric evaluation of the two instruments proceeded from EFA to reflective measurement-model assessment. The instrument-specific EFA, reliability, convergent-validity, and discriminant-validity procedures and criteria are described in "Work life quality instrument" section and "Professional ethics instrument" section. Common method bias was assessed using the procedures described in "Common method bias" section.

Finally, the structural model was evaluated using bootstrapping with 5,000 subsamples and bias-corrected confidence intervals. Hypotheses were assessed using standardized path coefficients, p-values, confidence intervals, and f² effect sizes. R² values were used to quantify explained variance, while blindfolding-based Q² values were used to assess in-sample predictive relevance. SRMR and NFI were reported as approximate model-fit indices. The structural model constituted the primary evidence for hypothesis testing, whereas group comparisons and correlations were treated as supplementary findings.

### Ethical considerations

All procedures conducted in this study adhered to the ethical standards outlined by the Declaration of Helsinki and were approved by the Research Ethics Committee of Tabriz University of Medical Sciences (*Ethics Code*: *IR.TBZMED.REC.1398.1295*). Prior to data collection, participants were fully informed about the purpose, scope, and voluntary nature of the research. A written informed consent form was distributed to all respondents, outlining their right to withdraw at any stage without consequence, as well as assurances of confidentiality and anonymity in data handling and reporting.

## Results

### Demographic profiles

From the 230 questionnaires distributed among occupational health professionals in East Azerbaijan Province, 30 responses were excluded due to incompleteness or inconsistencies. Thus, the final dataset consisted of 200 valid responses, resulting in an effective response rate of 86.9%. As shown in Table 3, the demographic characteristics of the respondents indicate a balanced yet diverse sample. The gender distribution was relatively balanced, with a slightly higher proportion of female participants (53.5%) than male participants (46.5%). The dominant age group was 20–30 years, representing 63.5% of the total sample, followed by those aged 31–40 years (27.5%). Regarding marital status, 55.5% were married, while 44.5% were single. In terms of professional background, most participants had 1 to 10 years of work experience (82.5%), while only 3.0% had more than 20 years of experience. Educational attainment showed that 71.5% held a bachelor’s degree, while 28.5% had postgraduate qualifications. Additionally, 60.5% had graduated less than 5 years ago, and 52.0% of the respondents had completed their education at top-tier medical universities in Iran.

**Table 3:** Demographic characteristics of respondents

Variable	Category	Frequency (*n*)	Percentage (%)
Gender	Female	107	53.5
	Male	93	46.5
Age group (years)	20–30	127	63.5
	31–40	55	27.5
	> 40	18	9.0
Marital status	Married	111	55.5
	Single	89	44.5
Work experience (years)	1–10	165	82.5
	11–20	29	14.5
	> 20	6	3.0
Education level	Bachelor’s degree	143	71.5
	Master’s or PhD	57	28.5
Monthly income (IRR)	Low	76	38.0
	Medium	95	47.5
	High	29	14.5
Years since graduation	< 5 years	121	60.5
	≥ 5 years	79	39.5
University type	Tier 1 medical universities	104	52.0
	Tier 2 or 3 medical universities	96	48.0

### Descriptive statistics

#### Descriptive statistics for the QWL dimensions

This section presents the descriptive statistics for the main constructs of the study: QWL and Professional Ethics. For QWL, the total raw scores ranged from 27 to 135, with a mean score of 82.00 (SD = 13.46), indicating a moderate overall perception of work life quality among respondents. Table 4 provides detailed descriptive statistics for the eight QWL dimensions, including the number of items, minimum and maximum possible scores, means and standard deviations, 95% confidence intervals, and standardized mean scores. When standardized scores were used to account for differences in the number of items across dimensions, Safe Work Environment had the highest mean score (56.87), whereas Growth Opportunities had the lowest (46.87).

**Table 4:** Descriptive statistics for QWL dimensions

Dimension	No. of Items	Min	Max	Mean (SD)	95% CI	Standardized Mean (SD)
Fair Compensation	3	3	15	9.14 (2.23)	8.82–9.45	51.16 (18.62)
Safe Work Environment	3	3	15	9.82 (1.88)	9.56–10.08	56.87 (15.68)
Growth Opportunities	3	3	15	8.62 (2.43)	8.28–8.96	46.87 (20.31)
Organizational Constitutionalism	4	4	20	12.13 (2.69)	11.75–12.51	50.84 (16.82)
Social Relevance	3	3	15	9.24 (2.01)	8.96–9.52	52.04 (16.78)
Work-Life Balance	3	3	15	8.66 (2.18)	8.35–8.96	47.16 (18.21)
Social Integration	4	4	20	11.73 (2.67)	11.35–12.10	48.31 (16.72)
Human Capabilities Development	4	4	20	12.73 (2.58)	12.36–13.09	54.56 (16.17)
Total QWL Score	27	27	135	82.00 (13.46)	80.21–83.96	51.00 (12.46)

For the Professional Ethics construct, total raw scores ranged from 29 to 145, with a mean of 76.43 (SD = 9.63). Based on the standardized scores, Justice and Confidentiality had the highest mean score (85.29), whereas Service Quality had the lowest (67.75). Table 5 presents the descriptive results for each of the six PE dimensions, following the same format.

**Table 5:** Descriptive statistics for Professional Ethics (PE) dimensions

Dimension	No. of Items	Min	Max	Mean (SD)	95% CI	Standardized Mean (SD)
Service Quality	3	3	15	11.13 (1.45)	10.92–11.34	67.75 (12.14)
Professional Responsibility	8	8	40	32.08 (4.26)	31.49–32.67	75.26 (13.32)
Professional Autonomy	4	4	20	16.64 (2.23)	16.32–16.95	79.00 (13.94)
Professional Excellence	3	3	15	12.28 (1.73)	12.04–12.52	77.37 (14.43)
Justice and Confidentiality	6	6	30	26.47 (3.13)	26.03–26.92	85.29 (13.08)
Trustworthiness	5	5	25	19.78 (2.52)	19.43–20.13	73.92 (12.63)
Total Professional Ethics Score	29	29	145	76.43 (9.63)	74.96–77.90	76.43 (9.63)

#### Assumption testing for parametric analyses

Prior to conducting independent-samples t-tests and one-way ANOVA, the assumptions of normality and homogeneity of variances were assessed to ensure the appropriateness of parametric statistical methods. Normality of the QWL and Professional Ethics scores across demographic subgroups was evaluated using the Shapiro–Wilk test, while the Levene’s test was used to examine homogeneity of variances. As summarized in Table 6, the Shapiro–Wilk test results indicated that the distribution of QWL and Professional Ethics scores did not significantly deviate from normality across all subgroups (All *p* > 0.05).

**Table 6:** Shapiro–wilk and Levene’s test results for QWL and professional ethics

Variable	Demographic Factor	Shapiro–Wilk *p*-value (QWL)	Shapiro–Wilk *p*-value (Ethics)	Levene’s Test *p*-value (QWL)	Levene’s Test *p*-value (Ethics)
Gender	Male vs. Female	0.267	0.312	0.373	0.289
Marital Status	Married vs. Single	0.198	0.277	0.446	0.390
Education Level	Bachelor vs. Postgrad	0.244	0.318	0.407	0.264
Income Level	High/ Moderate/ Low	0.296	0.332	0.278	0.338
Work Experience	1–10 / 11–20 / > 20 yrs.	0.204	0.240	0.343	0.216
Age Group	20–30 / 31–40 / > 40 yrs.	0.222	0.251	0.385	0.299
Graduation Year	< 5 yrs. / ≥ 5 yrs.	0.309	0.358	0.198	0.276
Secondary Job	Yes vs. No	0.271	0.349	0.369	0.415

### Supplementary group comparison analyses

#### Independent-samples t-test

Independent-samples t-tests were conducted to examine differences in QWL and Professional Ethics across gender and marital status. Levene’s test confirmed homogeneity of variances for all comparisons (*p* > 0.05). As shown in Table 7, a statistically significant gender difference was observed for QWL scores, with male participants reporting slightly higher QWL scores than female participants (*p* = 0.045). However, no significant gender difference was found for Professional Ethics scores (*p* = 0.329). For marital status, QWL scores were slightly higher among married participants but did not reach statistical significance (*p* = 0.077). However, married participants reported significantly higher Professional Ethics scores than single participants (*p* = 0.038).

**Table 7:** Independent-samples t-test results for gender and marital status

Variable	Group	*N*	Mean	SD	t	df	*p*-value
QWL	Male	93	82.94	15.86	2.02	198	0.045
	Female	107	81.34	10.97			
Professional Ethics	Male	93	76.72	9.94	0.98	198	0.329
	Female	107	76.13	9.29			
QWL	Married	111	82.71	13.92	1.78	198	0.077
	Single	89	81.12	12.87			
Professional Ethics	Married	111	76.78	9.33	2.09	198	0.038
	Single	89	75.91	9.87			

#### One-way ANOVA

As shown in Table 8, statistically significant differences in QWL were observed by education level (*p* = 0.034), income level (*p* = 0.017), and work experience (*p* = 0.042). Differences in QWL by age group (*p* = 0.089), graduation year (*p* = 0.285), and secondary job status (*p* = 0.212) were not statistically significant. For Professional Ethics, statistically significant differences were observed by education level (*p* = 0.041) and work experience (*p* = 0.048), whereas differences by income level (*p* = 0.072), age group (*p* = 0.361), graduation year (*p* = 0.392), and secondary job status (*p* = 0.229) were not statistically significant.

**Table 8:** One-way ANOVA results for QWL and professional ethics by demographic factors

Variable	Demographic Factor	F	df	*p*-value
QWL	Education Level	4.57	1,198	0.034
	Income Level	4.15	2,197	0.017
	Age Group	2.19	2,197	0.089
	Work Experience	3.29	2,197	0.042
	Graduation Year	1.15	2,197	0.285
	Secondary Job	1.57	1,198	0.212
Professional Ethics	Education Level	2.91	1,198	0.041
	Income Level	2.64	2,197	0.072
	Age Group	1.02	2,197	0.361
	Work Experience	2.78	2,197	0.048
	Graduation Year	0.94	2,197	0.392
	Secondary Job	1.46	1,198	0.229

### Measurement model evaluation

#### Main evaluation

To ensure the reliability and validity of the constructs used in this study, the measurement model was assessed prior to evaluating the structural model, following the standard PLS-SEM procedures. The model was examined for internal consistency reliability, convergent validity, and discriminant validity using SmartPLS 4.0.

To improve consistency across the measurement, hypothesis, and structural model sections, a consolidated measurement table was added (Table 9). This table reports the final construct labels, number of retained items, Cronbach’s alpha, composite reliability, AVE, standardized factor-loading range, and whether each construct was included in the structural model.

**Table 9:** Consolidated measurement properties of the study constructs

Construct	No. of items	Cronbach’s alpha	Composite reliability	AVE	Factor-loading range	Included in structural model
Fair Compensation	3	0.78	0.85	0.64	0.75–0.84	No
Safe Work Environment	3	0.74	0.84	0.62	0.73–0.82	Yes
Growth Opportunities	3	0.76	0.83	0.66	0.74–0.81	No
Organizational Constitutionalism	3	0.79	0.86	0.60	0.72–0.80	No
Work-Life Balance	3	0.78	0.85	0.61	0.71–0.79	No
Social Relevance	3	0.77	0.84	0.59	0.70–0.78	No
Social Integration	4	0.84	0.89	0.68	0.76–0.86	Yes
Human Capabilities Development	4	0.82	0.88	0.63	0.77–0.88	Yes
Professional Responsibility	8	0.88	0.92	0.72	0.78–0.89	Yes
Professional Autonomy	4	0.85	0.90	0.67	0.79–0.87	Yes
Trustworthiness	5	0.79	0.86	0.70	0.73–0.84	Yes
Professional Excellence	3	0.81	0.88	0.65	0.75–0.83	No
Service Quality	3	0.80	0.87	0.66	0.74–0.82	No
Justice and Confidentiality	6	0.83	0.89	0.69	0.75–0.85	No

#### Internal consistency reliability and convergent validity

Internal consistency was evaluated using Cronbach’s alpha and CR. As shown in Table 10, all constructs demonstrated acceptable to excellent reliability, with Cronbach’s alpha values ranging from 0.74 to 0.90 and CR values exceeding the threshold of 0.70 for all constructs. In terms of convergent validity, all AVE values were above the recommended minimum of 0.50, ranging from 0.62 to 0.74. This suggests that each construct explained more than half of the variance of its observed indicators, supporting convergent validity. For example, the construct Responsibility had an AVE of 0.74, indicating that its items were strongly representative of the underlying concept.

**Table 10:** Internal consistency and convergent validity of constructs

Construct	Cronbach’s Alpha	Composite Reliability	AVE
Fair Compensation	0.78	0.85	0.66
Safe Work Environment	0.74	0.84	0.64
Growth Opportunities	0.76	0.83	0.62
Social Integration	0.84	0.89	0.67
Human Capabilities Development	0.82	0.88	0.65
Professional Autonomy	0.85	0.90	0.71
Professional Responsibility	0.88	0.92	0.74
Trustworthiness	0.79	0.86	0.62
Professional Excellence	0.81	0.88	0.66
Quality of Work Life	0.90	0.93	0.72
Professional Ethics	0.87	0.91	0.69

#### Discriminant validity

Discriminant validity was assessed using two criteria: the Fornell-Larcker criterion and the Heterotrait-Monotrait Ratio (HTMT). According to the Fornell-Larcker criterion, the square root of AVE for each construct was greater than its correlations with all other constructs, suggesting acceptable discriminant validity. However, as recommended in recent literature, the HTMT criterion was also employed due to its greater sensitivity. As shown in Table 11, all HTMT values ranged between 0.53 and 0.78, well below the conservative cutoff of 0.85. For instance, the HTMT between QWL and Professional Ethics was 0.66, indicating sufficient discriminant validity between these two higher-order constructs.

**Table 11:** HTMT values among key constructs

	Quality of Work Life	Professional Responsibility	Professional Autonomy	Trustworthiness	Professional Ethics
Quality of Work Life	−	0.61	0.55	0.53	0.66
Professional Responsibility	−	−	0.67	0.59	0.78
Professional Autonomy	−	−	−	0.62	0.73
Trustworthiness	−	−	−	−	0.71
Total Professional Ethics	−	−	−	−	−

#### Factor loadings

**Fig. 2:**
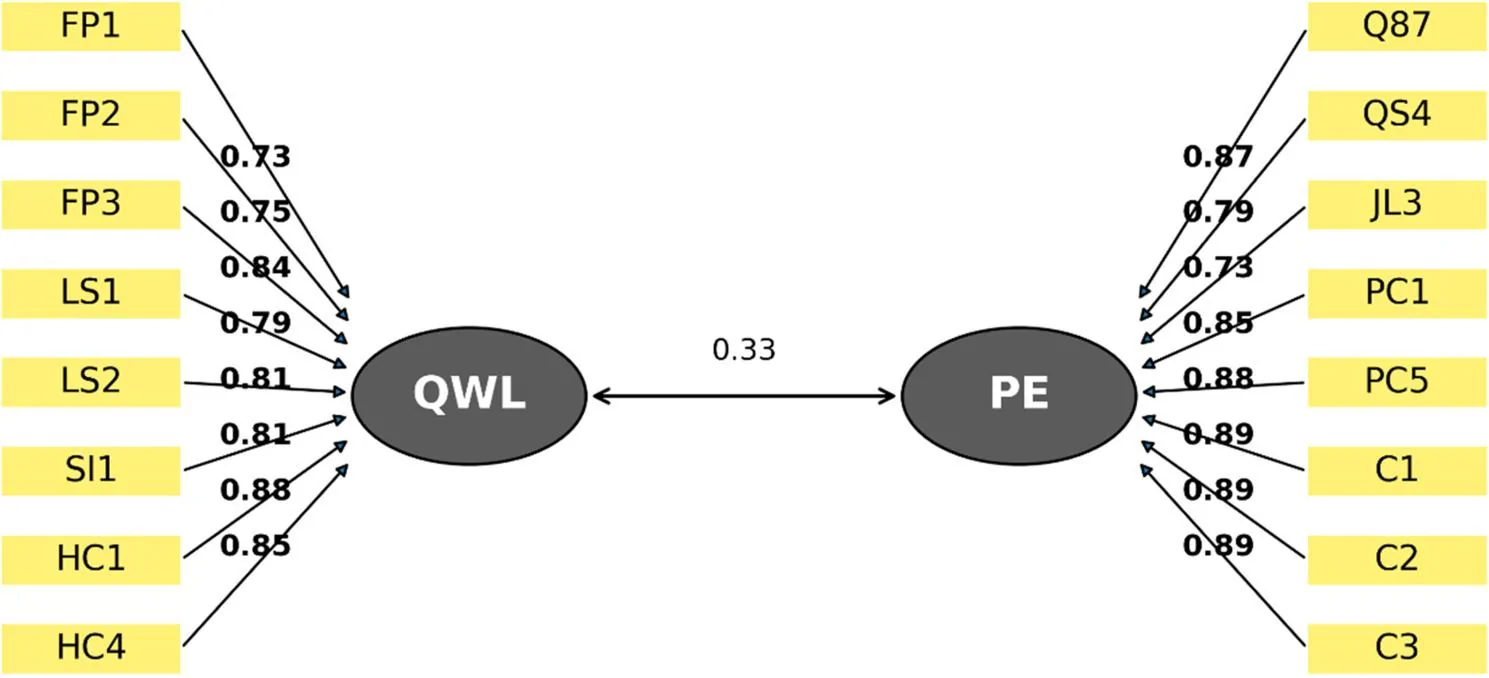
Measurement model with standardized loadings

All standardized factor loadings were statistically significant (*p* < 0.001). Across the consolidated measurement model presented in Table 11, the loadings ranged from 0.70 to 0.89, meeting the accepted criterion for indicator reliability. The subset of indicators displayed in Fig. 2 had loadings ranging from 0.73 to 0.89.

These findings confirm that each indicator adequately reflects its underlying construct within the QWL and Professional Ethics dimensions. The outer model structure visually summarizes these standardized relationships, thereby validating the robustness and appropriateness of the retained items for structural analysis.

#### Common method bias assessment

To assess the potential impact of CMB, Harman’s single-factor test was conducted (Table 12). The results indicated that the first unrotated factor accounted for 33.2% of the total variance, which is below the commonly used threshold of 40%. This finding suggests that common method bias was unlikely to substantially distort the results.

**Table 12:** Harman’s single-factor test and VIF values

Criterion	Value
Harman’s Single Factor Variance	33.2%
Minimum VIF	1.21
Maximum VIF	2.47
Threshold for CMB Concern	< 40% variance
Threshold for Multicollinearity	VIF < 3.3

In addition, all VIF values in the measurement model were below 3.3, ranging from 1.21 to 2.47. These results provide further diagnostic evidence against serious multicollinearity or common method bias concerns. However, given the single-source, self-report, cross-sectional design of the study, these procedures were interpreted as partial diagnostic checks rather than definitive evidence that common method bias was absent.

### Structural model assessment and hypothesis testing

Because the main objective of this study was to test a SET-based structural model, the PLS-SEM results were considered the primary basis for hypothesis testing, while correlation and group-comparison analyses were interpreted as preliminary and supplementary findings.

After confirming the adequacy of the measurement model, the structural model was assessed to test the nine directional hypotheses proposed in this study. Standardized path coefficients, t-values, *p*-values, bias-corrected confidence intervals, and f² effect sizes were examined using bootstrapping with 5,000 subsamples. The results of the structural model are presented in Table 13.

**Table 13:** Structural model results for hypothesis testing

Hypothesis	Structural path	β	t-value	*p*-value	95% CI (BC)	f²	Decision
H1	Human Capabilities Development → Professional Responsibility	0.298	4.872	< 0.001	[0.172, 0.418]	0.098	Supported
H2	Human Capabilities Development → Professional Autonomy	0.312	5.124	< 0.001	[0.188, 0.432]	0.112	Supported
H3	Human Capabilities Development → Trustworthiness	0.128	2.103	0.036	[0.009, 0.244]	0.017	Supported
H4	Safe Work Environment → Professional Responsibility	0.265	4.201	< 0.001	[0.141, 0.388]	0.078	Supported
H5	Safe Work Environment → Professional Autonomy	0.218	3.567	< 0.001	[0.098, 0.336]	0.052	Supported
H6	Safe Work Environment → Trustworthiness	0.125	2.045	0.041	[0.006, 0.242]	0.016	Supported
H7	Social Integration → Professional Responsibility	0.276	4.456	< 0.001	[0.154, 0.398]	0.085	Supported
H8	Social Integration → Professional Autonomy	0.238	3.892	< 0.001	[0.116, 0.358]	0.062	Supported
H9	Social Integration → Trustworthiness	0.158	2.567	0.010	[0.036, 0.278]	0.026	Supported

*β* standardized path coefficient, *CI *bias-corrected bootstrap confidence interval, *f² *effect size. Bootstrapping was performed using 5,000 subsamples. Effect sizes were interpreted as small, medium, and large at 0.02, 0.15, and 0.35, respectively

The results showed that all hypothesized paths were positive and statistically significant. Human Capabilities Development was significantly associated with Professional Responsibility, Professional Autonomy, and Trustworthiness. Safe Work Environment was also significantly associated with all three professional ethics dimensions. Similarly, Social Integration showed significant positive associations with Professional Responsibility, Professional Autonomy, and Trustworthiness. Therefore, H1 to H9 were supported. However, the f² values indicate that most effects were small, with H3 and H6 falling below the conventional threshold for a small effect.

The explanatory power and predictive relevance of the model were assessed using R², adjusted R², and Q² values. The results are shown in Table 14.

**Table 14:** Explained variance and in-sample predictive relevance of the endogenous constructs

Endogenous construct	*R*²	Adjusted *R*²	Q²
Professional Responsibility	0.214	0.202	0.141
Professional Autonomy	0.186	0.174	0.119
Trustworthiness	0.082	0.068	0.048

*R*² represents the proportion of variance explained in each endogenous construct. Positive Q² values suggest some in-sample predictive relevance but should not be interpreted as evidence of strong out-of-sample predictive performance

The model explained 21.4% of the variance in Professional Responsibility, 18.6% in Professional Autonomy, and 8.2% in Trustworthiness. These R² values indicate modest explanatory capacity overall and particularly limited explanatory power for Trustworthiness. The positive but relatively small Q² values for Professional Responsibility (0.141), Professional Autonomy (0.119), and Trustworthiness (0.048) suggest limited in-sample predictive relevance, with the weakest predictive relevance observed for Trustworthiness. These values should not be interpreted as evidence of strong predictive accuracy.

Approximate model fit was also assessed using SRMR and NFI. As shown in Table 15, the SRMR value was below the recommended threshold of 0.08, and the NFI value exceeded the recommended threshold of 0.90, indicating acceptable approximate model fit.

**Table 15:** Approximate model fit indices

Fit index	Value	Recommended threshold	Status
SRMR	0.062	< 0.08	Acceptable
NFI	0.91	> 0.90	Acceptable

*SRMR *standardized root mean square residual, *NFI *normed fit index

### Preliminary correlation analysis

To examine the strength and direction of associations between dimensions of QWL and Professional Ethics, Pearson correlation analysis was conducted. The results in Table 16 show that the overall QWL score was significantly and positively correlated with the overall professional ethics score (*r* = 0.281, *p* < 0.01), indicating a modest positive relationship. This suggests that occupational health professionals who reported more favourable perceptions of their work environment and conditions also tended to report higher professional ethics scores.

**Table 16:** Pearson correlation matrix between QWL and professional ethics

QWL Dimensions	Service Quality	Professional Responsibility	Professional Autonomy	Professional Excellence	Justice and Confidentiality	Trustworthiness	Total Professional Ethics Score
Fair Compensation	0.109	0.190	0.192	0.134	0.077	-0.014	0.160
Safe Work Environment	0.215	0.282	0.234	0.042	0.127	0.140	0.236
Growth Opportunities	0.120	0.269	0.189	0.071	0.066	0.069	0.180
Organizational Constitutionalism	0.184	0.267	0.222	0.055	0.146	0.095	0.234
Social Relevance	0.193	0.229	0.217	0.055	0.089	0.108	0.203
Work-Life Balance	0.074	0.061	0.053	-0.071	-0.028	-0.013	0.015
Social Integration	0.218	0.293	0.256	0.164	0.160	0.173	0.290
Human Capabilities Development	0.230	0.317	0.330	0.078	0.107	0.142	0.267
Total QWL Score	0.235	0.338	0.299	0.096	0.133	0.125	0.281

Values represent Pearson correlation coefficients. The total QWL–total professional ethics correlation was *r* = 0.281. The strongest domain-level correlation was between Human Capabilities Development and Professional Autonomy (*r* = 0.330)

At the domain level, several QWL components, including Fair Compensation, Safe Work Environment, Human Capabilities Development, and Social Integration, were positively correlated with multiple dimensions of professional ethics, including Professional Responsibility, Professional Autonomy, and Trustworthiness. However, some QWL dimensions, such as Work-Life Balance, showed weak or non-significant relationships with ethical components.

The strongest domain-level correlation was observed between Human Capabilities Development and Respect for Professional Autonomy (*r* = 0.330, *p* < 0.01), while the weakest and non-significant relationship was found between Work-Life Balance and Professional Excellence (*r* = -0.071, *p* = 0.31).

## Discussion

### Interpretation of findings and comparison with prior studies

The findings reveal a selective rather than uniform pattern of association between Quality of Work Life and Professional Ethics. Human Capabilities Development, Safe Work Environment, and Social Integration were more consistently associated with Professional Responsibility and Professional Autonomy than with Trustworthiness. This pattern suggests that ethical outcomes closely embedded in everyday organizational interactions may be more responsive to perceived workplace conditions, whereas Trustworthiness may depend more strongly on personal dispositions, professional socialization, ethical leadership, or organizational climate. The modest associations, generally small effect sizes, and limited explained variance support a cautious interpretation and indicate that Quality of Work Life provides only a partial account of Professional Ethics outcomes. Although all paths to Trustworthiness were statistically significant, their effect sizes were generally very small. The effects of Human Capabilities Development and Safe Work Environment fell below the conventional threshold for a small effect, while the effect of Social Integration was small. This suggests that Trustworthiness may be shaped by additional individual, organizational, or professional factors not fully captured in the present model.

These findings are consistent with SET, which suggests that supportive, fair, and development-oriented work environments may be associated with positive professional attitudes and behaviours through mechanisms of reciprocity, perceived organizational care, and moral obligation. However, because both Quality of Work Life and Professional Ethics were assessed through self-report at a single time point, the observed associations may partly reflect shared method variance, social desirability, or general response tendencies. The cross-sectional design also prevents establishing temporal ordering; for example, professionals with stronger ethical orientations may perceive their workplace conditions more favourably. The findings should therefore be interpreted as concurrent associations consistent with Social Exchange Theory rather than evidence that Quality of Work Life causes changes in Professional Ethics.

From a SET perspective, QWL may function as an organizational resource that supports Professional Ethics through reciprocal employee–organization relationships [29]. However, the weak association between Social Integration and Trustworthiness suggests that this relational influence is limited. More specifically, Human Capabilities Development may represent organizational investment, Safe Work Environment may signal institutional care, and Social Integration may strengthen belonging and shared ethical norms [30–37]. Their comparatively stronger associations suggest that workplace-based resources may be more relevant to Professional Ethics than broader conditions such as Work-Life Balance [38], consistent with previous evidence on dignity, empowerment, and inclusive organizational environments [3, 39].

Beyond the organizational factors captured in the model, educational attainment also appeared relevant to ethical behaviour. The significant association between educational attainment and ethical behaviour is consistent with Dejean et al. [15] and Omidi et al. [40], both of whom reported that higher academic qualifications were associated with ethical sensitivity and decision-making. Kaya et al. [41] also found that education was related to ethical responsiveness and caring behaviour among nursing students, while Kazemi et al. [42] linked moral reasoning and ethical behaviour with professional learning and training. In the present study, this pattern is consistent with the observed association between Human Capabilities Development and Professional Autonomy, suggesting that professional growth may be particularly relevant to autonomy-related ethical conduct. Safe Work Environment and Social Integration showed modest associations with Professional Ethics outcomes [43]. consistent with evidence on safety climate and inclusive workplace cultures [39, 41, 44]. In contrast, Work-Life Balance showed limited relevance.

However, unlike Nayak et al. [8], who found that income significantly influenced ethical behaviour among public health professionals, our study did not reveal a statistically significant relationship between income levels and ethical scores. A possible explanation lies in the structural detachment between compensation systems and professional responsibility mechanisms within Iran’s public-sector employment. In these systems, ethical behaviour may be guided more by deontological standards embedded in organizational codes than by material gain. Similarly, while our analysis showed no significant gender-based differences in ethical conduct, contrary to findings by Kaya et al. [41], this may reflect uniform codes of ethical regulation, standardized job descriptions, and similar supervision levels among male and female professionals. Moreover, studies in similar collectivist cultures such as Dehghani et al. [45] suggest that communal work norms and peer responsibility may reduce the influence of individual characteristics such as gender on ethical decision-making.

Professional Ethics did not differ by gender or age, suggesting that institutional norms may outweigh basic demographic traits [13]. Married participants reported higher ethical scores, contrary to previous evidence, potentially reflecting culturally shaped responsibilities [14, 46]. Work experience was also positively associated with both QWL and ethical behaviour, which is consistent with Lee et al. [47] and Sha et al. [20], who indicated that professional maturity is linked to judgment, self-regulation, and moral engagement in complex workplace settings. Although the present study did not directly examine ethical leadership, the findings resonate with Baydeniz et al. [21], who highlighted that experienced professionals may contribute to ethical work climates when supported by transparent performance appraisals and value-driven workplace systems. In contrast, variables such as year of graduation and holding a second job did not show significant associations with QWL or ethics in this study. This differs from Khanian et al. [11], who noted that job multiplicity may reduce ethical consistency due to role conflict and burnout. However, such effects may be less salient in occupational health settings where job scopes are relatively well-defined and workloads are systematically distributed.

Overall, the findings provide modest, context-specific support for SET, with developmental, relational, and safety-related QWL dimensions showing greater relevance to Professional Ethics. Differences from prior evidence highlight the need for culturally sensitive research and workplace ethics initiatives.

### Scientific contributions and policy implications

This study offers several novel contributions to the interdisciplinary literature on professional ethics and QWL, particularly within the domain of WHS. While a growing body of research has examined ethical behaviour in professions such as nursing, education, and management, there is a notable scarcity of empirical studies focusing specifically on WHS professionals. A comprehensive review across major scholarly databases revealed that ethical behaviour among occupational health specialists remains a largely unexplored topic. As a result, many of the studies used for comparative discussion in this paper are drawn from adjacent fields. This contextual gap highlights the novelty of our research and underscores the need to expand ethical inquiry into WHS-related disciplines.

Theoretically, this study provides preliminary, context-specific evidence that selected Quality of Work Life dimensions are associated with particular dimensions of Professional Ethics in a manner consistent with Social Exchange Theory. The observed pattern suggests that developmental and relational resources may be more closely associated with the examined ethical outcomes than some structural or material conditions. However, the modest R² values indicate that Social Exchange Theory and the selected Quality of Work Life dimensions provide only a partial explanation of these outcomes. The findings should therefore be regarded as a cautious application of Social Exchange Theory rather than a comprehensive explanatory model of occupational ethics.

From a policy perspective, the findings provide context-specific insights that may be useful for organizational leaders, human resource practitioners, and regulatory bodies. Investment in capability development and inclusive social structures may be relevant to supporting ethical conduct, although the modest explanatory power of the model suggests that other individual and organizational factors should also be considered. Policymakers may therefore consider workplace-based strategies as one component of broader efforts to support ethical cultures, while recognizing that the present model explains only a limited proportion of variation in Professional Ethics. Moreover, the cultural and institutional specificity of the findings calls for context-sensitive ethics promotion strategies, especially in collectivist environments like Iran’s public health sector. In such settings, ethical engagement may be better sustained through institutional trustworthiness and shared norms rather than through individual traits or material inducements.

Overall, this study addresses an important gap in the empirical literature and provides a preliminary analytical framework for examining ethical conduct among WHS professionals, while recognizing that the model explains only a limited proportion of variance in some ethical dimensions, particularly Trustworthiness.

### Limitations and future research directions

Despite offering valuable insights into the relationship between Quality of Work Life and professional ethics, several limitations should be considered when interpreting the findings. First, the cross-sectional design restricts the ability to draw causal inferences; therefore, the observed associations should be interpreted as relational rather than directional or causal. As a result, it cannot be determined whether improvements in QWL lead to stronger ethical behaviour or whether ethically committed professionals perceive their work environment more positively. Future research employing longitudinal, or panel designs could better clarify the temporal ordering and potential causal pathways between these constructs.

Second, the study was geographically limited to occupational health professionals in Iran, which may constrain the generalizability of the findings. Cultural norms, regulatory frameworks, and institutional practices may shape both perceptions of QWL and ethical behaviour, meaning that the observed relationships may not fully transfer to other national or organizational contexts. Multi-site and cross-cultural studies are therefore recommended to assess the robustness of the proposed relationships across diverse settings and regulatory environments.

Third, reliance on self-reported data introduces the possibility of social desirability and common method biases, which may have influenced the observed associations between QWL and ethical constructs. Although procedural and statistical checks were applied, these checks should be interpreted as partial diagnostic evidence rather than definitive proof that common method bias was absent. Future studies could mitigate this limitation by integrating objective indicators, supervisor ratings, peer assessments, or qualitative observations to triangulate ethical behaviour and workplace conditions.

Finally, the present study did not explicitly incorporate organizational-level variables such as ethical leadership, organizational justice, or formal ethics training programs. The absence of these variables limits the ability to fully explain the mechanisms through which QWL may be linked to ethical behaviour. Future research should include these factors as potential mediators or moderators to develop a more comprehensive and explanatory model of ethical conduct in occupational health settings. Overall, these limitations indicate that the findings should be interpreted as context-specific associations rather than causal or universally generalizable conclusions. Addressing these limitations through longitudinal designs, multi-source data, cross-cultural samples, and broader theoretical models would enhance the explanatory power, generalizability, and practical relevance of future research on Quality of Work Life and professional ethics.

## Conclusion

This study examined the associations between selected Quality of Work Life dimensions and Professional Ethics among occupational health professionals in Iran, using Social Exchange Theory as the guiding framework. Although several structural paths were statistically significant, the model explained only 21.4% of the variance in Professional Responsibility, 18.6% in Professional Autonomy, and 8.2% in Trustworthiness. Accordingly, the findings indicate modest and partial associations rather than strong explanatory or predictive capacity. The particularly low explained variance for Trustworthiness suggests that additional individual, organizational, and professional factors should be incorporated into future models.

From a practical perspective, the findings suggest that improving developmental, psychosocial, and supportive work conditions may be associated with stronger ethical orientations among occupational health professionals; however, the modest R² values indicate that these factors explain only part of the variation in ethical outcomes. For policymakers and regulatory bodies in the WHS domain, these results may inform training strategies, ethical supervision, and organizational support systems, particularly in collectivist and hierarchical work cultures. However, due to the cross-sectional design and regional scope of the study, the findings should be interpreted as associative rather than causal. Future longitudinal and mixed-method studies are recommended to examine potential causal pathways and mediating mechanisms, such as ethical climate, perceived justice and confidentiality, and organizational identification.

## Supplementary Information

Supplementary Material 1.

Supplementary Material 2.

## Data Availability

The data that support the findings of this study are available from the corresponding author upon reasonable request.

## Appendix 1

**Table 17:** Examples of questionnaire items and conceptual dimensions

Construct	Conceptual Dimension	Sample Item (Example)
Quality of Work Life	Human Capabilities Development	“I have opportunities to develop my professional skills in my workplace.”
Quality of Work Life	Safe Work Environment	“My workplace provides a Safe Work Environment for performing my duties.”
Quality of Work Life	Social Integration	“I feel respected and socially supported by my colleagues.”
Professional Ethics	Responsibility	“I feel accountable for the ethical consequences of my professional decisions.”
Professional Ethics	Professional Autonomy	“I respect individuals’ rights to informed decision-making in health-related matters.”
Professional Ethics	Trustworthiness	“Others can rely on me to act ethically in my professional role.”
