# Supplementary material for: Quality of work life and professional ethics among occupational health professionals: a social exchange theory perspective
Source: BMC Psychol. 2026 Jul 22;14:1501. doi: 10.1186/s40359-026-05226-6 (PMC13679131; doi:10.1186/s40359-026-05226-6)
Supplement: Supplementary file 1 [file 40359_2026_5226_MOESM1_ESM.docx]

**Professional Ethics Questionnaire**

| Title | Question |
| --- | --- |
| Professional Excellence | PE1. I continuously strive to improve my professional knowledge and skills in occupational health practice. PE2. I apply up-to-date scientific principles and standards in performing my professional duties. PE3. I take responsibility for maintaining professional competence in my field. |
| Ethical Behaviour and Trust | ET1. I act honestly and transparently in all professional interactions. ET2. I avoid any behaviour that may undermine trust in occupational health practice. ET3. I fulfil my professional duties even when external supervision is limited. ET4. I maintain integrity in reporting safety and health information. ET5. I behave in a manner that strengthens colleagues’ and workers’ trust. |
| Quality of Services | QS1. I provide occupational health services with a high level of accuracy and care. QS2. I ensure that professional decisions prioritise workers’ health and safety. QS3. I strive to deliver services that meet accepted professional and ethical standards. |
| Human Dignity and Autonomy | HD1. I respect the dignity of workers regardless of their position or background. HD2. I respect individuals’ rights to informed decision-making regarding health matters. HD3. I avoid any form of discrimination in professional practice. HD4. I consider workers’ values and concerns when making professional judgments. |
| Accountability and Responsibility | AR1. I accept responsibility for the outcomes of my professional decisions. AR2. I report occupational health risks even when doing so may be inconvenient. AR3. I adhere to legal and professional obligations in my work. AR4. I respond appropriately to ethical dilemmas in the workplace. AR5. I am accountable for protecting workers’ health and safety. AR6. I take corrective action when professional errors occur. AR7. I prioritise ethical responsibility over personal benefit. AR8. I comply with professional codes of conduct in all work situations. |
| Justice and Confidentiality | JC1. I treat all workers fairly and impartially. JC2. I protect the confidentiality of workers’ health information. JC3. I do not misuse confidential information for personal or organisational gain. JC4. I ensure equal access to occupational health services. JC5. I handle sensitive information with professional discretion. JC6. I respect privacy principles in all occupational health activities. |

**Note:**

- The Professional Ethics Questionnaire was developed by the authors based on national professional ethics codes and relevant literature.
- The items represent the operational definitions of each ethical construct as described in the Methods section of the manuscript.
