# Supplementary material for: Quality of work life and professional ethics among occupational health professionals: a social exchange theory perspective
Source: BMC Psychol. 2026 Jul 22;14:1501. doi: 10.1186/s40359-026-05226-6 (PMC13679131; doi:10.1186/s40359-026-05226-6)
Supplement: Supplementary file 2 [file 40359_2026_5226_MOESM2_ESM.docx]

**STROBE checklist**

| Section | Item No | Recommendation | Page / Section in Manuscript |
| --- | --- | --- | --- |
| Title and Abstract | 1a | Indicate the study’s design | Abstract – Methods (“A cross-sectional survey…”) |
|  | 1b | Informative and balanced abstract | Entire Abstract |
| Introduction | 2 | Scientific background and rationale | Introduction, paragraphs 1–4 |
|  | 3 | Specific objectives / hypotheses | End of Introduction |
| Methods | 4 | Key elements of study design | Section 3.1 Research Design |
|  | 5 | Setting, location, dates | Section 3.2 Population and Sampling |
|  | 6a | Eligibility criteria and participant selection (cross-sectional) | Section 3.2 |
|  | 7 | Definitions of outcomes, predictors, confounders | Sections 3.3–3.3.3 |
|  | 8 | Data sources and measurement methods | Sections 3.3.1–3.3.4 |
|  | 9 | Addressing potential sources of bias | Section 3.3.4 (CMB & cultural adaptation) |
|  | 10 | Study size rationale | Section 3.2 (sample adequacy & PLS-SEM rules) |
|  | 11 | Handling of quantitative variables | Section 3.4 |
|  | 12a | Statistical methods | Section 3.4 |
|  | 12b | Subgroup and interaction analyses | Sections 4.3–4.3.2 |
|  | 12c | Handling of missing data | Section 3.4 (no missing data reported) |
|  | 12d | Sampling strategy (cross-sectional) | Section 3.2 |
|  | 12e | Sensitivity analyses | Section 3.4 (bootstrapping, robustness checks) |
| Results | 13a | Numbers at each study stage | Section 4.1 |
|  | 13b | Reasons for non-participation | Section 4.1 |
|  | 13c | Flow diagram consideration | Section 4.1 (textual reporting) |
|  | 14a | Participant characteristics | Tables 4–6 |
|  | 14b | Missing data per variable | Section 3.4 |
|  | 15 | Outcome data (cross-sectional) | Sections 4.2–4.5 |
|  | 16a | Unadjusted / adjusted estimates with precision | Sections 4.3–4.5 |
|  | 16b | Category boundaries | Sections 4.3–4.3.2 |
|  | 16c | Absolute risk translation (if relevant) | Not applicable (cross-sectional design) |
|  | 17 | Other analyses | Sections 4.3–4.5 |
| Discussion | 18 | Key results summary | Section 5.1 |
|  | 19 | Study limitations | Section 5.3 |
|  | 20 | Overall interpretation | Sections 5.1–5.2 |
|  | 21 | Generalisability | Section 5.3 |
| Other Information | 22 | Funding source and role | Funding section |

**Notes for Submission**

- All STROBE items relevant to cross-sectional studies are fully addressed.
- No item is missing.
- Items marked “not applicable” are explicitly justified by study design.
